# Supplementary material for: MVA Vectored Vaccines Encoding Rift Valley Fever Virus Glycoproteins Protect Mice against Lethal Challenge in the Absence of Neutralizing Antibody Responses
Source: Vaccines (Basel). 2020 Feb 12;8(1):82. doi: 10.3390/vaccines8010082 (PMC7157666; doi:10.3390/vaccines8010082)
Supplement: Supplementary file 1 [file vaccines-08-00082-s001.pdf]

vaccines

Supplementary Material

Supplementary Table S1. Primers used for inverse PCR reaction

Name	5′-3′ sequence	M-segment nucleotide positions (DQ380208.1)
end gn reverse	[Phos]GTGACACTGGTAATTTATCAG	1760-1739
startpb9 fwd:	TCCTACATACCATCTGCC	
endtpa reverse:	AGATCCTCTTCTGAATCG	
startgc fwd:	[Phos]TGTTCAGAACTGATTCAGG	2091-2109

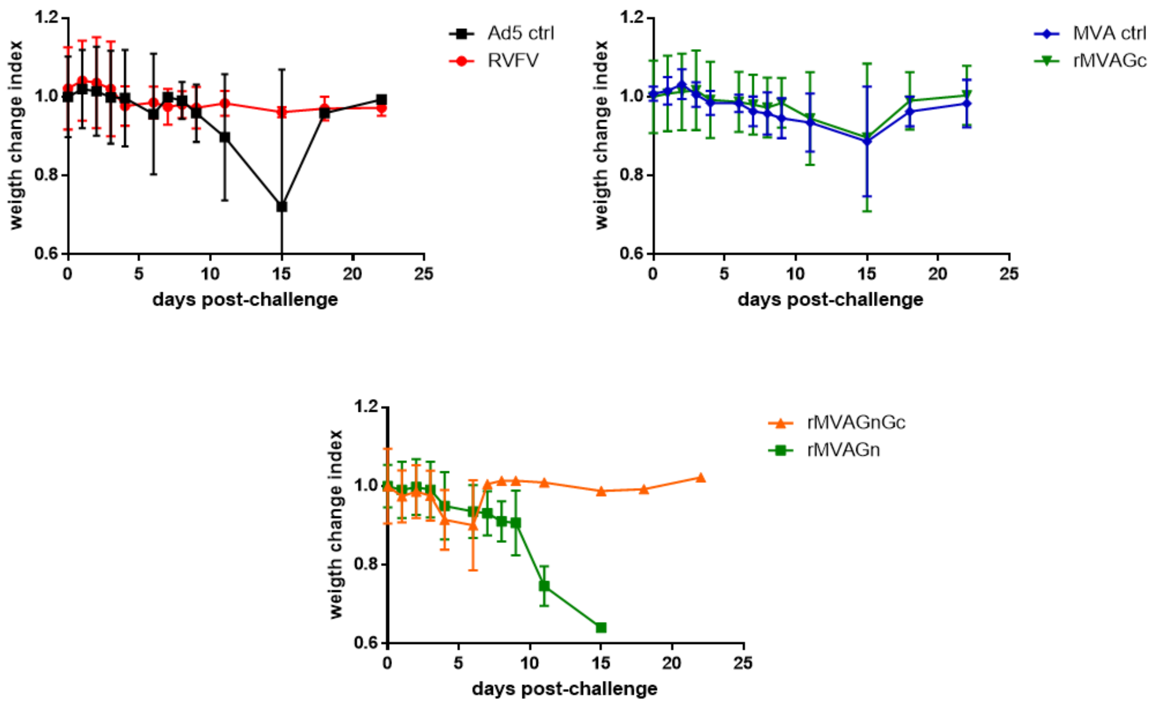

Supplementary Figure S1- Weight loss after passive transfer in mice

Amino acid sequence of eGn encoded by rMVAGn

MDAMKRGLCCVLLLCGAVFVSPSQEIHARFRRGSMAGIAMTVLPALAVFALAPVVFAEDPHL
RNRPGKGHNYIDGMTQEDATCKPVTYAGACSSFDVLLEKGKFPLFQ^{SYAHHRTLL}EAVHDTIIAKADP
PSCDLLSAHGNPCMKEKLVMKTHCPNNYQSAHHLNNDGKMASVKCPPKYELTEDCNFCRQM
TGASLKKGSYPLQDLFCQSSEDDGSKLKTKMKGVCEVGVQALKKCDGQLSTAHEVVPFAVFKNS
KKVYLDKLDLKTEENLLPDSFVCFEHKGQYKGTMDFGQTKRELKSFDISQCPKIGGHGSKKCTGD
AAFCSAYECTAQYANAYCSHANGSGIVQIQVSGVWKKPLCVGYERVVVKRELSAKPIQRVEPCT
TCITKCEPHGLVVRSTGFKISSAVACASGVCVTGSQSPSTEITLKYPGISQSSGGDIGVHMAHDDQS
VSSKIVAHCPPQDPCLVHDCIVCAHGLINYQCH**SYIPSAEKIIPNPLLGLD**

Amino acid sequence of Gc encoded by rMVAGc

MDAMKRGLCCVLLLCGAVFVSPSQEIHARFRRGSCSELIQASSRITTCSTEGVNTKCRLSGTALI
RAGSVGAEACLMLKGVKEDQTKFLKLKTVSSELSCREGQSYWTGSFSPKCLSSRRCHLVGECHVN
RCLSWRDNETSAEFSFVGESTTMRENKCFEQCGGWGCGCFNVNPSCLFVHTYLQSVRKEALRVF
NCIDWVHKLTLEITDFDGSVSTIDLGASSSRFTNWGSVSLSLDAEGISGSNSFSFIESPGKGYAIVDE
PFSEIPRQGFLGEIRCNSESSVLSAHESCLRAPNLI^{SYKPMIDQL}ECTTNLIDPFVVFERGSLPQTRNDKTF
AASKGNRGVQAFSKGSVQADLTLMFDNFEVDFVGAAVSCDAAFLNLTGCYSCNAGARVCLSITS
TGTGSLSAHNKDGSLHIVLPSENGTKDQCQILHFTVPEVEEEFMYSCDGDERPLLVKGTLIAIDPF
DDRREAGGESTVVNPKSGSWNFFDWFSGLMSWF^{GGPLKTILL}ICLYVALSIGLFFLLIYLGGTGLSKMW
LAATKKASRS**SYIPSAEKIIPNPLLGLD**

Supplementary Figure S2- Amino acid sequences of eGn and Gc encoded by the recombinant MVAs. **Bold**: N-terminal tPA leader sequence and C-terminal tag pb9/V5 epitope. Underlined: putative transmembrane-cytoplasmic tail of Gc. ^{Superscript}: class-I MHC peptides used in this work
